# 3,3-Bis(2-hy­droxy­eth­yl)-1-(4-nitro­benzo­yl)thio­urea: crystal structure, Hirshfeld surface analysis and computational study

**DOI:** 10.1107/S2056989019017328

**Published:** 2020-01-07

**Authors:** Sang Loon Tan, Mukesh M. Jotani, Edward R. T. Tiekink

**Affiliations:** a Research Centre for Crystalline Materials, School of Science and Technology, Sunway University, 47500 Bandar Sunway, Selangor Darul Ehsan, Malaysia; b Department of Physics, Bhavan’s Sheth R. A. College of Science, Ahmedabad, Gujarat 380001, India

**Keywords:** crystal structure, thio­urea, nitro group, hydrogen bonding, Hirshfeld surface analysis, computational chemistry

## Abstract

In the title compound, C_12_H_15_N_3_O_5_S, a tris­ubstituted thio­urea derivative, the central CN_2_S chromophore is almost planar (r.m.s. deviation = 0.018 Å) and the pendant hy­droxy­ethyl groups lie to either side of this plane. While to a first approximation the thione-S and carbonyl-O atoms lie to the same side of the mol­ecule, the S—C—N—C torsion angle of −47.8 (2)° indicates a considerable twist. As one of the hy­droxy­ethyl groups is orientated towards the thio­amide residue, an intra­molecular N—H⋯O hydrogen bond is formed which leads to an *S*(7) loop. A further twist in the mol­ecule is indicated by the dihedral angle of 65.87 (7)° between the planes through the CN_2_S chromophore and the 4-nitro­benzene ring. There is a close match between the experimental and gas-phase, geometry-optimized (DFT) mol­ecular structures. In the crystal, O—H⋯O and O—H⋯S hydrogen bonds give rise to supra­molecular layers propagating in the *ab* plane. The connections between layers to consolidate the three-dimensional architecture are of the type C—H⋯O, C—H⋯S and nitro-O⋯π. The nature of the supra­molecular association has been further analysed by a study of the calculated Hirshfeld surfaces, non-covalent inter­action plots and computational chemistry, all of which point to the significant influence and energy of stabilization provided by the conventional hydrogen bonds.

## Abstract 2

In the title tri-substituted thio­urea mol­ecule, a substantial twist is evident as seen in the dihedral angle of 65.92 (12)° between the planes through the CN_2_S residue and the 4-nitroaryl ring; an intra­molecular N—H⋯O hydrogen bond leading to an *S*(7) loop is noted. In the mol­ecular packing, O—H⋯O and O—H⋯S hydrogen bonds lead to supra­molecular layers propagating in the *ab* plane.

## Chemical context   

In addition to accepting C—H⋯O inter­actions, nitro groups are known to form nitro-N—O⋯π(ar­yl) inter­actions (Huang *et al.*, 2008 ▸) as well as participate as donors and acceptors in π-hole inter­actions (Bauzá *et al.*, 2014 ▸). Hence, when the title nitro-containing compound, (I), became available, a crystallographic analysis was undertaken. Compound (I) is an example of a tri-substituted thio­urea mol­ecule, H_2_NC(=S)NH_2_, whereby three of the four hydrogen atoms have been substituted to yield 4-NO_2_C_6_H_4_C(=O)N(H)C(=S)N(CH_2_CH_2_OH)_2_. Such *N*,*N*′-di(alk­yl/ar­yl)-*N*′-benz­oyl­thio­urea derivatives have a carbonyl group connected to the thio­urea framework and offer opportunities for rich coordination chemistry as these mol­ecules feature both hard (oxygen) and soft (sulfur) donor atoms along with nitro­gen donors and indeed, a variety of coordination modes have been observed. The neutral mol­ecule has been observed to coordinate in a monodentate-*S* mode (Gunasekaran, Ng *et al.*, 2012 ▸; Saeed *et al.*, 2014 ▸). In its deprotonated form, *O*-,*S*- chelation is often observed (Saeed *et al.*, 2014 ▸). There are a variety of motivations for investigating metal complexes of benzoyl­thio­urea derivatives such as for catalytic applications and for anion recognition (Zhang & Schreiner, 2009 ▸; Gunasekaran, Jerome *et al.*, 2012 ▸; Nishikawa, 2018 ▸). Over and above these considerations, there are continuing investigations into their biological potential, such as anti-microbial (Gemili *et al.*, 2017 ▸; Binzet *et al.*, 2018 ▸; Saeed *et al.*, 2018 ▸), anti-cancer (Peng *et al.*, 2016 ▸; Barolli *et al.*, 2017 ▸; Jeyalakshmi *et al.*, 2019 ▸) and anti-mycobacterium tuberculosis (Plutín *et al.*, 2016 ▸) agents. In a continuation of our on-going work on these mol­ecules and their metal complexes (Selvakumaran, Ng *et al.*, 2011 ▸; Selvakumaran, Karvembu *et al.*, 2011 ▸; Gunasekaran *et al.*, 2017 ▸; Tan, Azizan *et al.*, 2019 ▸), we now describe the synthesis, spectroscopic characterization and X-ray crystallographic investigation of (I). Further, an analysis of the calculated Hirshfeld surfaces, non-covalent inter­action plots as well as a computational chemistry study for (I) are described.
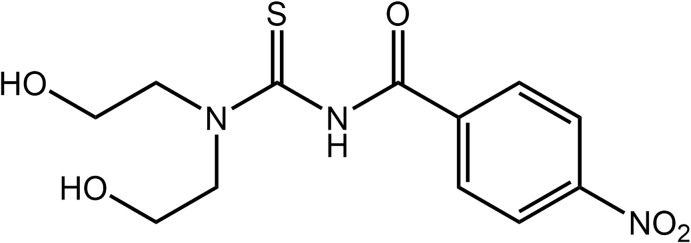


## Structural commentary   

**Figure 1:**
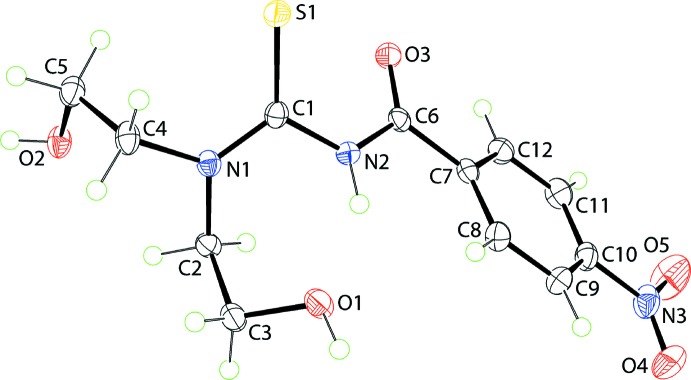
The mol­ecular structure of (I) showing the atom-labelling scheme and displacement ellipsoids at the 70% probability level.

Selected geometrical data for (I), Fig. 1 ▸, are given in Table 1 ▸. The key feature of the structure is that it is a tri-substituted thio­urea mol­ecule with one of the nitro­gen atoms having a benzoyl residue and the other bearing two hy­droxy­ethyl groups. An approximate *syn* relationship is established between the thione-S and carbonyl-O atoms. Even though they lie to the same side of the mol­ecule, the S1—C1—N2—C6 torsion angle of −47.8 (2)° is consistent with a significant twist in the mol­ecule about the C1—N2 bond; the O3—C6—N2—C1 torsion angle is −3.6 (2)°.

The hy­droxy­ethyl groups lie to either side of the CN_2_S plane (r.m.s. deviation = 0.017 Å). Crucially, the O1-hydroxy­ethyl group is folded towards the thio­amide residue, which allows for the formation of an intra­molecular N2—H⋯O1 hydrogen bond and an *S*(7) loop, Table 2 ▸. That the mol­ecule is highly twisted is evidenced by the dihedral angle of 65.87 (7)° between the CN_2_S atoms and the terminal C7–C12 aryl ring. From Table 1 ▸, it is apparent that the C1—N1 bond length is considerably shorter than C1—N2, indicating delocalization of π-electron density over the S1—C1—N1 atoms. However, the large twist for the C1—N2 bond mentioned above does not allow significant delocalization to extend to atoms C1, N1 and C6. The expected trends relating to the nature of the bonds about the quaternary-C1 atom are seen in the bond angles about that atom. Thus, the angles subtended by the formally doubly bonded S1 atom are appreciably wider. Finally, the nitro group is effectively co-planar with the aryl ring to which it is attached, as seen in the O4—N3—C10—C9 torsion angle of 5.2 (2)°.

## Gas-phase theoretical structure   

**Figure 2:**
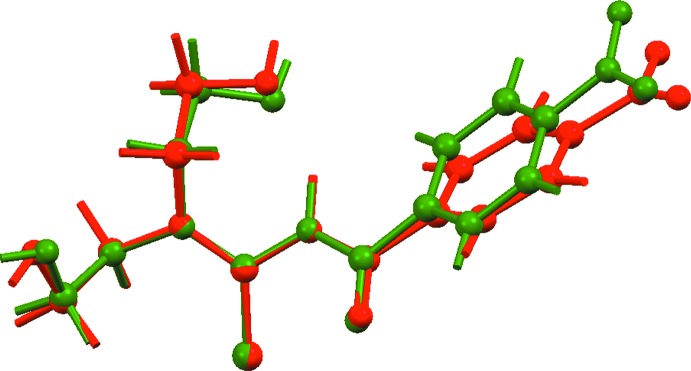
Overlay diagram for the experimental (green image) and geometry-optimized (red) mol­ecules of (I). The mol­ecules have been overlapped so the S=C—N—C=O fragments are coincident.

With the aid of a long-range corrected wB97XD density functional with Grimme’s D2 dispersion model (Chai & Head-Gordon, 2008 ▸) and coupled with Pople’s 6-311+G(*d*,*p*) basis set (Petersson *et al.*, 1988 ▸), as implemented in *Gaussian16* (Frisch *et al.*, 2016 ▸), the gas-phase geometry-optimized structure of (I) was calculated. As confirmed through a frequency analysis with zero imaginary frequency, the local minimum structure in the gas-phase was located in this study. The experimental and theoretical structures are superimposed (Macrae *et al.*, 2006 ▸) in Fig. 2 ▸. The analysis shows that there are only minor differences between the mol­ecules with the r.m.s. deviation between the conformations being only 0.015 Å. The derived inter­atomic data for the geometry-optimized structure are included in Table 1 ▸ from which it can be seen there is a close correlation between the experimental and calculated geometries.

It is evident that the only major differences between the experimental and geometry-optimized structures relate to some of the torsion angles. Thus, the most significant conformational difference is evidenced by a nearly 13° difference in the O3—C6—N2—C1 torsion angles, *i.e*. −3.6 (2)° (X-ray) *versus* −16.5° (calculation), indicating a greater deviation from the *anti*-disposition in the optimized structure. Also, the N1—C2—C3—O1 and N1—C4—C5—O2 torsion angles are close to symmetric in the optimized structure *cf*. the experimental structure. Similar trends were noted in analogous calculations performed on the 4-methyl analogue (Tan, Azizan *et al.*, 2019 ▸).

## Supra­molecular features   

**Figure 3:**
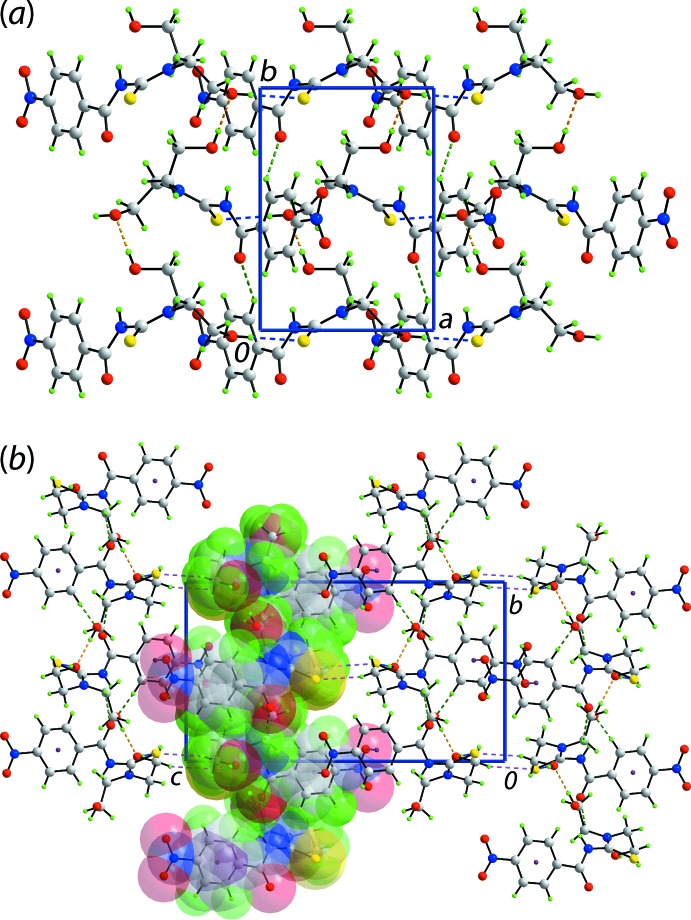
Views of the mol­ecular packing in (I): (*a*) supra­molecular layer in the *ab* plane sustained by hy­droxy-O—H⋯O(hy­droxy) and hy­droxy-O—H⋯S(thione) hydrogen bonds and (*b*) view of the unit-cell contents in a projection down the *a* axis, highlighting the methyl­ene-C—H⋯O(carbon­yl), methyl­ene-C—H⋯S(thione) and nitro-O⋯π(ar­yl) connections between layers; one layer is represented in space-filling mode. The O—H⋯O, O—H⋯S, C—H⋯O, C—H⋯S and N—O⋯π inter­actions are shown as orange, blue, green, pink and purple dashed lines, respectively.

In the crystal of (I), O1—H1*O*⋯O2 hydrogen bonds (Table 2 ▸) lead to a helical chain propagating along the *b-*axis direction, with adjacent mol­ecules related by the 2_1_ screw axis. The O2—H2*O*⋯S1 hydrogen bonding serves to cross-link translationally related chains along the *a* axis to form a supra­molecular layer in the *ab* plane, Fig. 3 ▸(*a*). The layers are connected into a three-dimensional architecture by methyl­ene-C—H⋯O(carbon­yl), methyl­ene-C—H⋯S(thione) and comparatively rare nitro-O⋯π(ar­yl) contacts, Fig. 3 ▸(*b*).

## Hirshfeld surface analysis   

**Figure 4:**
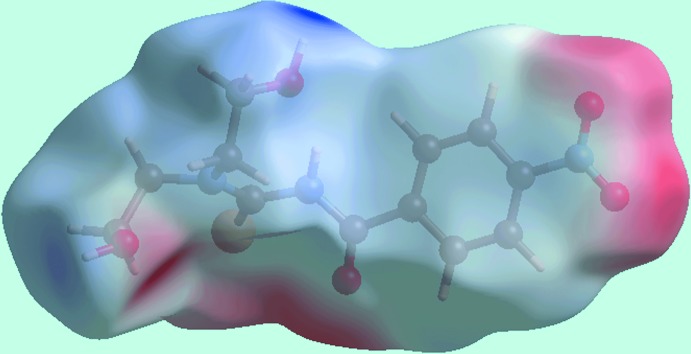
A view of the Hirshfeld surface mapped over the calculated electrostatic potential for (I). The red and blue regions represent negative and positive electrostatic potentials, respectively. The potentials were calculated using the STO-3G basis set at Hartree–Fock level of theory over a range of ±0.18 atomic units.

**Figure 5:**
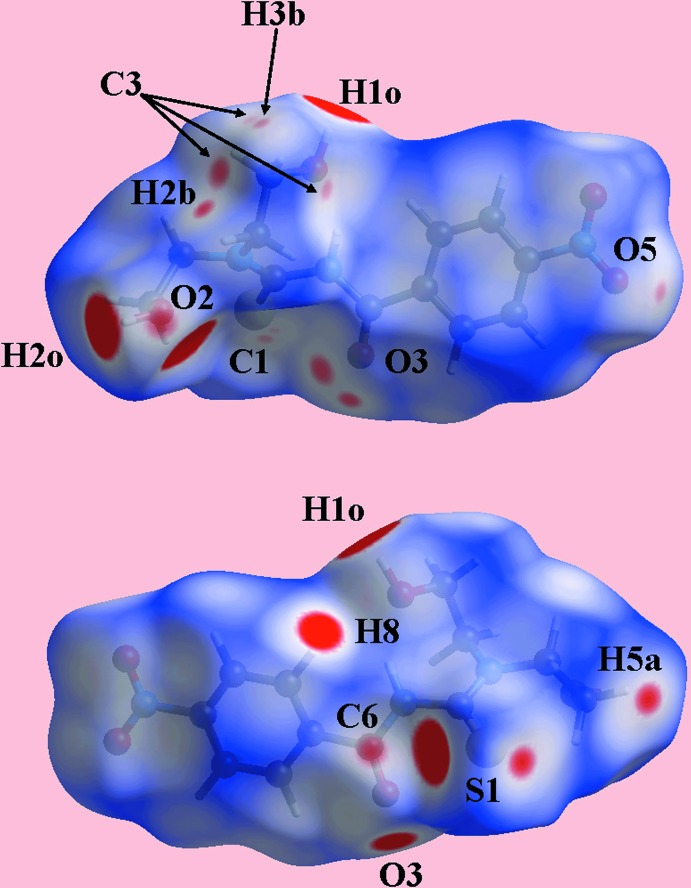
Two views of the Hirshfeld surface mapped over *d*_norm_ for (I) in the range −0.127 to +1.259 arbitrary units.

**Figure 6:**
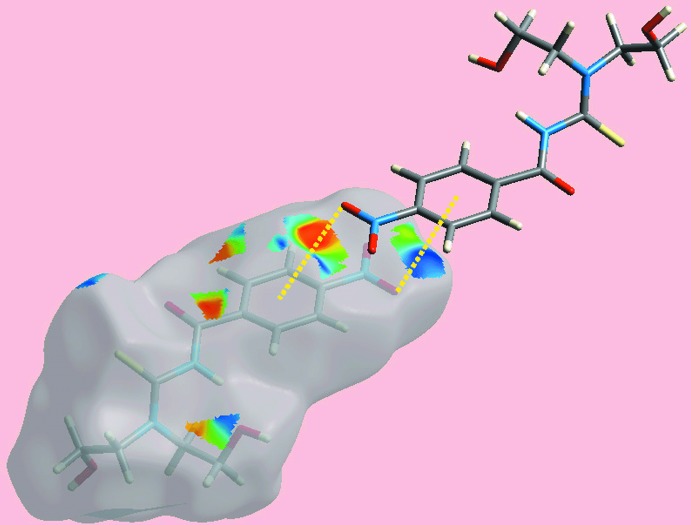
A view of the Hirshfeld surfaces mapped with the shape-index property for (I), highlighting the inter­molecular N—O⋯π(ar­yl) inter­actions through yellow dotted lines.

Using *Crystal Explorer 17* (Turner *et al.*, 2017 ▸) and established procedures (Tan, Jotani *et al.*, 2019 ▸), the Hirshfeld surfaces and two-dimensional fingerprint plots (full and decomposed) for (I) were calculated. In the Hirshfeld surface mapped over electrostatic potential in Fig. 4 ▸, the donors and acceptors of the conventional O—H⋯O and O—H⋯S hydrogen bonds and C—H⋯O contacts appear as blue (positive potential) and red (negative potential) regions, respectively. The bright-red spots near the participating atoms in the Hirshfeld surface mapped over *d*_norm_ in Fig. 5 ▸ also give indications of these inter­molecular inter­actions. Additional diminutive red spots near the methyl­ene-H2*B* and H5*A*, thione-S1 and carbonyl-O3 atoms are indicative of weaker C—H⋯S and C—H⋯O inter­actions, Table 2 ▸. Further, the presence of faint-red spots near the ethyl-C3 and nitro-O5 atoms on the surface indicate C—H⋯O contacts in the packing involving the nitro substit­uent. The other faint-red spots appearing in Fig. 5 ▸ indicate the presence of short inter­atomic contacts as summarized in Table 3 ▸. The influence of the nitro group is also seen in the nitro-O4⋯π(C7–C12) inter­action, illustrated through yellow dotted lines in Fig. 6 ▸.

The enrichment ratio (ER) descriptor, which is derived from the analysis of the Hirshfeld surface (Jelsch *et al.*, 2014 ▸), was also employed to analyse the inter­molecular contacts in the crystal of (I). The ER(*X*, *Y*) reflects the relative likelihood of the formation of *X*-to-*Y* inter­actions in a crystal, *i.e*. the ratio between the proportion of actual contacts in a crystal to the theoretical proportion of random contacts. Data for (I) are given in Table 4 ▸. The enrichment ratios greater than unity for the atom pairs (O, H) and, in particular, (S, H), are consistent with the relatively high likelihood for the formation of the O—H⋯O and O—H⋯S hydrogen bonds in the crystal of (I). It is also evident that the value greater than unity for (C, O) arises from the nitro-O⋯π(ar­yl) contacts.

**Figure 7:**
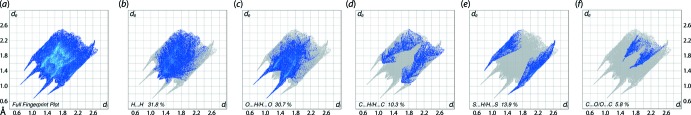
(*a*) A comparison of the full two-dimensional fingerprint plot for (I) and those delineated into (*b*) H⋯H, (*c*) O⋯H/H⋯O, (*d*) C⋯H/H⋯C, (*e*) S⋯H/H⋯S and (*f*) C⋯O/O⋯C contacts.

The overall fingerprint plots for (I) and those delineated into H⋯H, O⋯H/H⋯O, C⋯H/H⋯C, S⋯H/H⋯S and C⋯O/O⋯C contacts are illustrated in Fig. 7 ▸(*a*)–(*f*), respectively, with a summary of the percentage contributions from the various contacts given in Table 5 ▸. The greatest contribution to the overall surface is from H⋯H contacts and this is closely followed by O⋯H/H⋯O contacts, as viewed by the pair of long spikes at *d*_e_ + *d*_i_ ∼1.8 Å in Fig. 7 ▸(*c*). The prominent features in Fig. 7 ▸(*d*) reflect the significant C⋯H/H⋯C contacts evident in the packing, Tables 2 ▸ and 3 ▸. The significant percentage contribution from S⋯H/H⋯S contacts reflects the presence of O—H⋯S hydrogen bonding and is apparent through the appearance of asymmetric spikes of different shapes at *d*_e_ + *d*_i_ ∼2.1 Å in the fingerprint plot of Fig. 7 ▸(*e*). The 5.8% contribution from C⋯O/O⋯C contacts and the aforementioned ER value of 1.66 clearly indicate the significance of the nitro-N—O⋯π inter­action upon the packing; this inter­action is reflected in the pair of short spikes *d*_e_ + *d*_i_ ∼3.0 Å, Fig. 7 ▸(*f*).

## Computational chemistry   

The energy calculations were performed using DFT-wB97XD/aug-cc-pVTZ (Woon & Dunning, 1993 ▸) to evaluate the strength of the inter­molecular O—H⋯O, O—H⋯S and C—H⋯O inter­actions between the respective pairs of mol­ecules. The BSSE corrected inter­action energies (*E* ^BSSE^_int_) are listed in Table 6 ▸. From these data, it is clear the O—H⋯O hydrogen bond has the greatest inter­action energy, followed by C—H⋯O and O—H⋯S. These results reflect those reported recently for the 4-methyl analogue (Tan, Azizan *et al.*, 2019 ▸).

**Figure 8:**
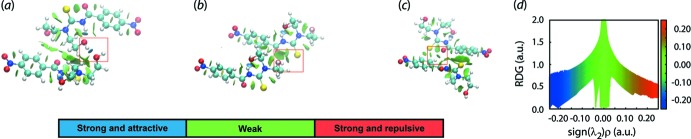
The non-covalent inter­action (NCI) plots for the dimeric aggregates in (I) sustained by (*a*) O—H⋯O, (*b*) O—H⋯S and (*c*) C—H⋯O inter­actions (highlighted in boxes) and (*d*) plot of RDG versus sign(λ^2^)*ρ*(r). The gradient cut-off is set at 0.4 and the colour scale is −0.03 < ρ < 0.03 atomic units.

The non-covalent inter­action plots generated by calculations performed with *NCIPLOT* (Johnson *et al.*, 2010 ▸) provide complementary results for the inter­action energies. Thus, the pairs of mol­ecules associated with each of the energies tabulated in Table 6 ▸ were subjected to calculation as this provides a useful visualization index corresponding to the strength of any non-covalent inter­actions through a red–blue–green colour scheme on the isosurface. Thus, a blue coloration is indicative of a strong attractive inter­action, green indicates a weak inter­action while red is indicative of a strong repulsive inter­action (Contreras-García *et al.*, 2011 ▸). As seen from Fig. 8 ▸, the O—H⋯O inter­action is clearly strong and attractive, while each of O—H⋯S and C—H⋯O are less so.

**Figure 9:**
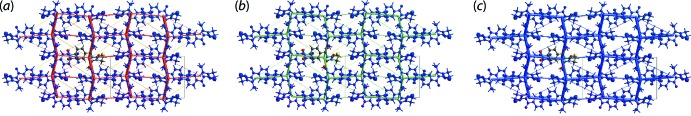
The energy framework diagrams for (I) showing (*a*) *E*_electrostatic_ (red cylinders), (*b*) *E*_dispersion_ (green cylinders) and (*c*) *E*_total_ (blue cylinders), viewed along the *a* axis. The frameworks were adjusted to the same scale factor of 50 with a cut-off value of 2.39 kcal/mol within 2 × 2 × 2 unit cells. The corresponding cylinder radii are proportional to the relative magnitude of the energies.

From the aforementioned, the mol­ecular packing is clearly governed by directional hydrogen bonding between mol­ecules. The simulated energy frameworks (Turner *et al.*, 2017 ▸) were calculated to compare the topology of the inter­molecular inter­actions in the crystal of (I). An analysis of the resultant energy frameworks is shown in Fig. 9 ▸ and reveals the crystal of (I) is mainly stabilized by electrostatic and dispersive forces. The total electrostatic energy (*E*_electrostatic_) of all pairwise inter­actions sums to −45.89 kcal/mol, while the total dispersion energy term (*E*_dispersion_) computes to −51.51 kcal/mol.

## Database survey   

**Figure 10:**
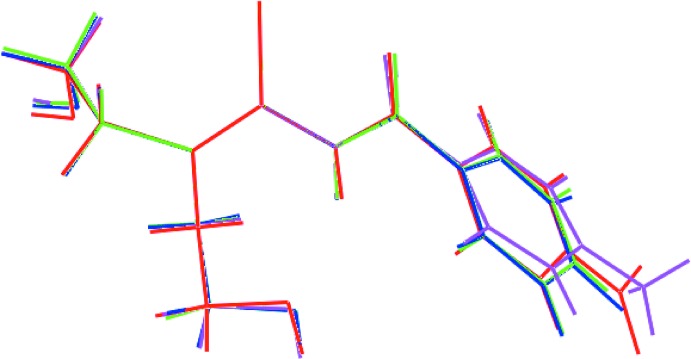
An overlay diagram of the four known structures of general formula 4-*Y*C_6_H_4_C(=O)N(H)C(=S)N(CH_2_CH_2_OH)_2_: *Y* = NO_2_ (I) red image, *Y* = H (green), *Y* = F (blue) and *Y* = Me (pink). The mol­ecules are overlapped so the central CN_2_S residues are coincident.

There are three literature precedents to (I), *i.e*. mol­ecules of the general formula 4-*Y*C_6_H_4_C(=O)N(H)C(=S)N(CH_2_CH_2_OH)_2_, namely *Y* = H, which has been reported twice (Koch *et al.*, 1995 ▸; Cornejo *et al.*, 2005 ▸), *Y* = F (Hennig *et al.*, 2009 ▸) and *Y* = Me (Tan, Azizan *et al.*, 2019 ▸). As seen in the overlay diagram of Fig. 10 ▸, whereby the central CN_2_S residues are overlapped, there is a very close coincidence in the mol­ecular structures. The differences in conformation are most conveniently expressed in terms of the dihedral angles formed between the central CN_2_S chromophore and pendant aryl ring, *i.e*. 65.92 (12), 68.96 (12), 69.51 (8) and 72.15 (10)° for (I) and *Y* = H, F and Me, respectively.

The mol­ecular packing in the crystals is also very similar with the formation of the intra­molecular thio­amide-N—H⋯O(hy­droxy) hydrogen bond as well as the inter­molecular hy­droxy-O—H⋯O(hy­droxy) hydrogen and hy­droxy-O—H⋯S(thione) hydrogen bonding, leading to a supra­molecular layer in each case.

## Synthesis and crystallization   

Synthesis of (I): an excess of thionyl chloride (Merck) was mixed with 4-nitro­benzoic acid (Merck, 1 mmol) and the resulting solution was refluxed until a pale-yellow solution was obtained. The excess thionyl chloride was removed on a water bath, leaving only 4-nitro­benzoyl chloride, which is a yellow, viscous liquid. Ammonium thio­cyanate (Fisher, 1 mmol) was added to an acetone (30 ml) solution of 4-nitro­benzoyl chloride (1 mmol). The solution turned yellow after stirring for 2 h. The white precipitate (ammonium chloride) was isolated upon filtration and to the yellow filtrate, bis­(hy­droxy­eth­yl)amine (Acros, 1 mmol) was carefully added followed by stirring for 1 h. Upon the addition of di­chloro­methane (50 ml), a yellow precipitate was obtained, which was collected by filtration. Recrystallization was from its hot acetone solution yielding pale-yellow blocks of (I) after slow evaporation. Yield 69%. M.p. (Hanon MP-450 melting point apparatus): 411.5–413.7 K. IR (Bruker Vertex 70v FT–IR spectrophotometer, cm^−1^): 3277 (*br*, *ν*OH), 3170 (*br*, *ν*NH), 3077 (*w*, *ν*CH_aro_), 2973–2882 (*w*, *ν*CH), 1692 (*s*, *ν*C=O), 1538 (*s*, *ν*N=O_asym_), 1524 (*s*, *ν*C=C), 1343 (*s*, *ν*N=O_sym_), 1270 (*s*, *ν*C—N), 1053 (*s*, *ν*C=S), 734 (*s*, *δ*CH). UV (Shimadzu UV 3600 Plus UV–vis spectrophotometer; ethanol:aceto­nitrile (1/1): *λ*_max_ nm (log *∊*) 366.4 (4.16), 301.6 (4.88), 271.2 (5.00), 205.8 (5.14).

The pyrolytic process (Perkin Elmer STA 6000 Simultaneous Thermogravimetric Analyzer) for (I) showed the liberation of NO_2_, equivalent a 15% weight loss, in the first stage in the range 194 and 222°C. This was followed by the liberation of a benzene mol­ecule, corresponding to 29% weight loss, between 222 and 282°C, whereas the subsequent stages involve the pyrolysis of CO (282 to 360°C) and OH (360 to 496°C) corresponding to 15 and 11% weight loss, respectively. Gradual weight loss continued beyond 800°C.

## Refinement   

Crystal data, data collection and structure refinement details are summarized in Table 7 ▸. Carbon-bound H atoms were placed in calculated positions (C—H = 0.95–0.99 Å) and were included in the refinement in the riding-model approximation, with *U*_iso_(H) set to 1.2*U*_eq_(C). The O- and N-bound H atoms were located from a difference map and refined with O—H and N—H = 0.84±0.01 and 0.88±0.01 Å, respectively, and with *U*_iso_(H) = 1.5*U*_eq_(O) and 1.2*U*_eq_(N).

## Supplementary Material

Crystal structure: contains datablock(s) I, global. DOI: 10.1107/S2056989019017328/hb7880sup1.cif

Structure factors: contains datablock(s) I. DOI: 10.1107/S2056989019017328/hb7880Isup2.hkl

CCDC reference: 1919879

Additional supporting information:  crystallographic information; 3D view; checkCIF report

## Figures and Tables

**Table 1:** Selected geometric parameters (Å, °) for (I) determined experimentally (X-ray) and from theory (DFT)

Parameter	X-ray	Theory
C1=S1	1.6777 (16)	1.668
C1—N1	1.334 (2)	1.366
C1—N2	1.4038 (19)	1.410
C6—O3	1.2156 (18)	1.219
C6—N2	1.3771 (19)	1.388
		
S1—C1—N1	123.83 (12)	124.7
S1—C1—N2	121.89 (11)	121.8
N1—C1—N2	114.23 (13)	113.4
O3—C6—N2	123.57 (14)	124.3
O3—C6—C7	121.17 (13)	121.1
N2—C6—C7	115.20 (13)	114.5
		
S1—C1—N2—C6	−47.8 (2)	−44.6
S1—C1—N1—C2	173.80 (11)	167.8
S1—C1—N1—C4	−8.0 (2)	−7.2
O3—C6—N2—C1	−3.6 (2)	−16.5
O3—C6—C7—C8	163.29 (15)	152.7
N1—C2—C3—O1	−62.76 (17)	−69.3
N1—C4—C5—O2	57.76 (17)	68.4

**Table 2:** Hydrogen-bond geometry (Å, °) *Cg*1 is the centroid of the (C7–C12) ring.

*D*—H⋯*A*	*D*—H	H⋯*A*	*D*⋯*A*	*D*—H⋯*A*
N2—H2*N*⋯O1	0.87 (1)	1.88 (1)	2.6749 (17)	151 (1)
O1—H1*O*⋯O2^i^	0.84 (2)	1.87 (2)	2.7075 (17)	176 (2)
O2—H2*O*⋯S1^ii^	0.84 (1)	2.33 (1)	3.1724 (12)	175 (2)
C2—H2*B*⋯O3^i^	0.99	2.53	3.2305 (18)	127
C5—H5*A*⋯S1^iii^	0.99	2.77	3.4915 (17)	130
C8—H8⋯O3^iv^	0.95	2.36	3.2147 (19)	150
N3—O4⋯*Cg*1^v^	1.22 (1)	3.63 (1)	3.6927 (16)	83 (1)

Symmetry codes: (i) 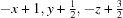; (ii) 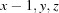; (iii) 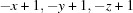; (iv) 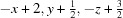; (v) 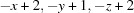.

**Table 3:** A summary of short inter­atomic contacts (Å) in (I)^*a*^

Contact	Distance	Symmetry operation
H1*O*⋯H2*O*	2.23	1 − *x*, 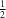 + *y*, 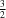 − *z*
C1⋯C3	3.368 (2)	1 − *x*, − 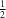 + *y*, 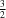 − *z*
C1⋯H3*B*	2.71	1 − *x*, − 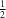 + *y*, 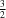 − *z*
C3⋯O3	3.0819 (19)	1 − *x*, 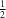 + *y*, 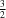 − *z*
C3⋯O5	3.168 (2)	2 − *x*, 1 − *y*, 1 − *z*
H3*B*⋯O5	2.65	2 − *x*, 1 − *y*, 1 − *z*
C5⋯H1*O*	2.61	1 − *x*, − 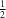 + *y*, 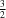 − *z*
C6⋯O2	3.0924 (18)	1 + *x*, *y*, *z*
C6⋯H2*O*	2.81	1 + *x*, *y*, *z*

Note: (*a*) The inter­atomic distances are calculated in *Crystal Explorer 17* (Turner *et al.*, 2017 ▸) whereby the *X*—H bond lengths are adjusted to their neutron values.

**Table 4:** Enrichment ratios for (I)

Parameter	Ratio
H⋯H	0.88
C⋯H	0.85
O⋯H	1.26
S⋯H	1.66
C⋯O	1.34

**Table 5:** Percentage contributions of inter­atomic contacts to the Hirshfeld surface for (I)

Contact	Percentage contribution
H⋯H	31.8
O⋯H/H⋯O	30.7
C⋯H/H⋯C	10.3
S⋯H/H⋯S	13.9
C⋯O/O⋯C	5.8
N⋯H/H⋯N	1.9
O⋯O	1.6
C⋯N/N⋯C	1.5
C⋯C	1.3
N⋯O/O⋯N	0.9
N⋯N	0.3

**Table 6:** Summary of inter­action energies (kcal mol^−1^) calculated for several directional contacts in (I)

Contact	*E*_tot_
O1—H1*O*⋯O2	−14.04
O2—H2*O*⋯S1	−5.60
C8—H8⋯O3	−10.05

**Table 7:** Experimental details

Crystal data	
Chemical formula	C_12_H_15_N_3_O_5_S
*M*_r_	313.33
Crystal system, space group	Monoclinic, *P*2_1_/*c*
Temperature (K)	100
*a*, *b*, *c* (Å)	7.4203 (2), 10.3241 (3), 18.4191 (6)
β (°)	95.471 (2)
*V* (Å^3^)	1404.62 (7)
*Z*	4
Radiation type	Mo *K*α
μ (mm^−1^)	0.26
Crystal size (mm)	0.12 × 0.11 × 0.09

Data collection	
Diffractometer	Bruker *SMART* *APEX* diffractometer
Absorption correction	Multi-scan (*SADABS*; Sheldrick, 1996 ▸)
*T*_min_, *T*_max_	0.970, 0.977
No. of measured, independent and observed [*I* > 2σ(*I*)] reflections	13082, 3233, 2621
*R*_int_	0.041
(sin θ/λ)_max_ (Å^−1^)	0.650

Refinement	
*R*[*F* ^2^ > 2σ(*F* ^2^)], *wR*(*F* ^2^), *S*	0.037, 0.093, 1.04
No. of reflections	3233
No. of parameters	200
No. of restraints	3
H-atom treatment	H atoms treated by a mixture of independent and constrained refinement
Δρ_max_, Δρ_min_ (e Å^−3^)	0.33, −0.25

Computer programs: *SMART* and *SAINT* (Bruker, 2008 ▸), *SHELXS97* (Sheldrick, 2015*a* ▸), *SHELXL2014*/7 (Sheldrick, 2015*b* ▸), *ORTEP-3 for Windows* (Farrugia, 2012 ▸), *DIAMOND* (Brandenburg, 2006 ▸) and *publCIF* (Westrip, 2010 ▸).

## supplementary crystallographic information

### Crystal data

**Table:**

C_12_H_15_N_3_O_5_S	*F*(000) = 656
*M**_r_* = 313.33	*D*_x_ = 1.482 Mg m^−^^3^
Monoclinic, *P*2_1_/*c*	Mo *K*α radiation, λ = 0.71073 Å
*a* = 7.4203 (2) Å	Cell parameters from 2434 reflections
*b* = 10.3241 (3) Å	θ = 2.3–25.8°
*c* = 18.4191 (6) Å	µ = 0.26 mm^−^^1^
β = 95.471 (2)°	*T* = 100 K
*V* = 1404.62 (7) Å^3^	Block, pale yellow
*Z* = 4	0.12 × 0.11 × 0.09 mm

### Data collection

**Table:**

Bruker SMART APEX diffractometer	3233 independent reflections
Radiation source: fine-focus sealed tube	2621 reflections with *I* > 2σ(*I*)
Graphite monochromator	*R*_int_ = 0.041
φ and ω scans	θ_max_ = 27.5°, θ_min_ = 2.2°
Absorption correction: multi-scan (SADABS; Sheldrick, 1996)	*h* = −9→9
*T*_min_ = 0.970, *T*_max_ = 0.977	*k* = −13→13
13082 measured reflections	*l* = −23→23

### Refinement

**Table:**

Refinement on *F*^2^	Primary atom site location: structure-invariant direct methods
Least-squares matrix: full	Secondary atom site location: difference Fourier map
*R*[*F*^2^ > 2σ(*F*^2^)] = 0.037	Hydrogen site location: mixed
*wR*(*F*^2^) = 0.093	H atoms treated by a mixture of independent and constrained refinement
*S* = 1.04	*w* = 1/[σ^2^(*F*_o_^2^) + (0.042*P*)^2^ + 0.3083*P*] where *P* = (*F*_o_^2^ + 2*F*_c_^2^)/3
3233 reflections	(Δ/σ)_max_ = 0.001
200 parameters	Δρ_max_ = 0.33 e Å^−^^3^
3 restraints	Δρ_min_ = −0.25 e Å^−^^3^

### Special details

**Table:**

Geometry. All esds (except the esd in the dihedral angle between two l.s. planes) are estimated using the full covariance matrix. The cell esds are taken into account individually in the estimation of esds in distances, angles and torsion angles; correlations between esds in cell parameters are only used when they are defined by crystal symmetry. An approximate (isotropic) treatment of cell esds is used for estimating esds involving l.s. planes.

### Fractional atomic coordinates and isotropic or equivalent isotropic displacement parameters (Å^2^)

**Table:**

	*x*	*y*	*z*	*U*_iso_*/*U*_eq_	
S1	0.74915 (5)	0.45567 (4)	0.59498 (2)	0.02217 (12)	
O1	0.72148 (15)	0.75229 (11)	0.77993 (8)	0.0313 (3)	
H1O	0.760 (3)	0.8221 (13)	0.7992 (11)	0.047*	
O2	0.16441 (15)	0.47542 (11)	0.65347 (6)	0.0219 (3)	
H2O	0.0554 (16)	0.466 (2)	0.6367 (12)	0.051 (7)*	
O3	0.88241 (15)	0.29890 (10)	0.73714 (6)	0.0221 (3)	
O4	1.35804 (19)	0.56698 (14)	1.06330 (7)	0.0407 (4)	
O5	1.3728 (2)	0.35951 (15)	1.07036 (8)	0.0538 (4)	
N1	0.52846 (17)	0.57554 (12)	0.67890 (7)	0.0168 (3)	
N2	0.80071 (17)	0.51231 (12)	0.73839 (7)	0.0174 (3)	
H2N	0.801 (2)	0.5824 (12)	0.7648 (8)	0.021*	
N3	1.3235 (2)	0.45902 (16)	1.03874 (8)	0.0300 (4)	
C1	0.6860 (2)	0.51602 (14)	0.67325 (8)	0.0167 (3)	
C2	0.4669 (2)	0.61682 (14)	0.74939 (8)	0.0181 (3)	
H2A	0.5141	0.5555	0.7879	0.022*	
H2B	0.3330	0.6135	0.7461	0.022*	
C3	0.5291 (2)	0.75230 (15)	0.77061 (10)	0.0232 (4)	
H3A	0.4853	0.8146	0.7320	0.028*	
H3B	0.4800	0.7783	0.8166	0.028*	
C4	0.4055 (2)	0.60787 (16)	0.61391 (8)	0.0211 (3)	
H4A	0.4779	0.6236	0.5723	0.025*	
H4B	0.3402	0.6889	0.6232	0.025*	
C5	0.2690 (2)	0.50167 (17)	0.59362 (9)	0.0224 (3)	
H5A	0.1876	0.5288	0.5506	0.027*	
H5B	0.3329	0.4220	0.5807	0.027*	
C6	0.89516 (19)	0.40521 (14)	0.76552 (8)	0.0163 (3)	
C7	1.0107 (2)	0.42635 (14)	0.83615 (8)	0.0172 (3)	
C8	1.0674 (2)	0.54755 (15)	0.86196 (9)	0.0205 (3)	
H8	1.0348	0.6228	0.8340	0.025*	
C9	1.1712 (2)	0.55922 (16)	0.92821 (9)	0.0229 (4)	
H9	1.2105	0.6417	0.9463	0.027*	
C10	1.2160 (2)	0.44763 (17)	0.96720 (9)	0.0225 (4)	
C11	1.1652 (2)	0.32570 (16)	0.94236 (9)	0.0249 (4)	
H11	1.2000	0.2507	0.9702	0.030*	
C12	1.0628 (2)	0.31546 (15)	0.87614 (9)	0.0222 (3)	
H12	1.0275	0.2325	0.8576	0.027*	

### Atomic displacement parameters (Å^2^)

**Table:**

	*U*^11^	*U*^22^	*U*^33^	*U*^12^	*U*^13^	*U*^23^
S1	0.0180 (2)	0.0320 (2)	0.0167 (2)	0.00001 (16)	0.00252 (15)	−0.00347 (16)
O1	0.0161 (6)	0.0216 (6)	0.0562 (9)	−0.0018 (5)	0.0031 (6)	−0.0148 (6)
O2	0.0150 (6)	0.0302 (6)	0.0202 (6)	−0.0025 (5)	0.0007 (5)	0.0039 (5)
O3	0.0206 (6)	0.0177 (6)	0.0274 (6)	0.0012 (4)	−0.0010 (5)	−0.0045 (5)
O4	0.0425 (8)	0.0469 (8)	0.0303 (7)	−0.0041 (7)	−0.0090 (6)	−0.0105 (6)
O5	0.0680 (11)	0.0522 (9)	0.0356 (8)	−0.0006 (8)	−0.0231 (8)	0.0156 (7)
N1	0.0158 (6)	0.0181 (6)	0.0166 (6)	0.0005 (5)	0.0015 (5)	0.0018 (5)
N2	0.0171 (7)	0.0172 (6)	0.0177 (6)	0.0020 (5)	0.0000 (5)	−0.0032 (5)
N3	0.0241 (8)	0.0442 (10)	0.0212 (7)	−0.0019 (7)	−0.0010 (6)	0.0024 (7)
C1	0.0150 (7)	0.0157 (7)	0.0190 (8)	−0.0025 (6)	0.0003 (6)	0.0011 (6)
C2	0.0167 (8)	0.0183 (7)	0.0198 (7)	0.0008 (6)	0.0035 (6)	−0.0007 (6)
C3	0.0162 (8)	0.0208 (8)	0.0324 (9)	0.0023 (6)	0.0009 (7)	−0.0043 (7)
C4	0.0178 (8)	0.0258 (8)	0.0191 (8)	0.0014 (6)	−0.0007 (6)	0.0069 (7)
C5	0.0169 (8)	0.0334 (9)	0.0167 (8)	−0.0003 (7)	0.0010 (6)	0.0013 (7)
C6	0.0125 (7)	0.0175 (7)	0.0193 (7)	−0.0003 (6)	0.0041 (6)	−0.0001 (6)
C7	0.0136 (7)	0.0198 (8)	0.0187 (8)	0.0012 (6)	0.0038 (6)	0.0008 (6)
C8	0.0188 (8)	0.0194 (8)	0.0228 (8)	−0.0006 (6)	0.0003 (6)	0.0034 (6)
C9	0.0215 (8)	0.0223 (8)	0.0243 (8)	−0.0039 (6)	−0.0001 (7)	−0.0029 (7)
C10	0.0174 (8)	0.0329 (9)	0.0169 (8)	0.0008 (7)	−0.0005 (6)	0.0005 (7)
C11	0.0257 (9)	0.0238 (8)	0.0248 (8)	0.0049 (7)	0.0004 (7)	0.0077 (7)
C12	0.0224 (8)	0.0185 (8)	0.0254 (8)	0.0027 (6)	0.0008 (7)	−0.0001 (6)

### Geometric parameters (Å, º)

**Table:**

S1—C1	1.6777 (16)	C3—H3A	0.9900
O1—C3	1.4218 (19)	C3—H3B	0.9900
O1—H1O	0.843 (10)	C4—C5	1.515 (2)
O2—C5	1.4331 (19)	C4—H4A	0.9900
O2—H2O	0.844 (10)	C4—H4B	0.9900
O3—C6	1.2156 (18)	C5—H5A	0.9900
O4—N3	1.221 (2)	C5—H5B	0.9900
O5—N3	1.220 (2)	C6—C7	1.504 (2)
N1—C1	1.334 (2)	C7—C8	1.389 (2)
N1—C4	1.4723 (19)	C7—C12	1.396 (2)
N1—C2	1.4796 (19)	C8—C9	1.385 (2)
N2—C6	1.3771 (19)	C8—H8	0.9500
N2—C1	1.4038 (19)	C9—C10	1.381 (2)
N2—H2N	0.871 (9)	C9—H9	0.9500
N3—C10	1.479 (2)	C10—C11	1.379 (2)
C2—C3	1.512 (2)	C11—C12	1.378 (2)
C2—H2A	0.9900	C11—H11	0.9500
C2—H2B	0.9900	C12—H12	0.9500
			
C3—O1—H1O	110.5 (15)	C5—C4—H4B	109.1
C5—O2—H2O	108.2 (16)	H4A—C4—H4B	107.8
C1—N1—C4	121.38 (13)	O2—C5—C4	110.21 (13)
C1—N1—C2	123.19 (13)	O2—C5—H5A	109.6
C4—N1—C2	115.41 (12)	C4—C5—H5A	109.6
C6—N2—C1	125.31 (13)	O2—C5—H5B	109.6
C6—N2—H2N	119.3 (11)	C4—C5—H5B	109.6
C1—N2—H2N	115.1 (11)	H5A—C5—H5B	108.1
O5—N3—O4	123.33 (16)	O3—C6—N2	123.57 (14)
O5—N3—C10	118.05 (15)	O3—C6—C7	121.17 (13)
O4—N3—C10	118.63 (15)	N2—C6—C7	115.20 (13)
N1—C1—N2	114.23 (13)	C8—C7—C12	119.91 (14)
N1—C1—S1	123.83 (12)	C8—C7—C6	123.75 (14)
N2—C1—S1	121.89 (11)	C12—C7—C6	116.34 (14)
N1—C2—C3	112.40 (13)	C9—C8—C7	120.31 (15)
N1—C2—H2A	109.1	C9—C8—H8	119.8
C3—C2—H2A	109.1	C7—C8—H8	119.8
N1—C2—H2B	109.1	C10—C9—C8	118.14 (15)
C3—C2—H2B	109.1	C10—C9—H9	120.9
H2A—C2—H2B	107.9	C8—C9—H9	120.9
O1—C3—C2	108.01 (12)	C11—C10—C9	122.97 (15)
O1—C3—H3A	110.1	C11—C10—N3	118.37 (15)
C2—C3—H3A	110.1	C9—C10—N3	118.66 (15)
O1—C3—H3B	110.1	C12—C11—C10	118.26 (15)
C2—C3—H3B	110.1	C12—C11—H11	120.9
H3A—C3—H3B	108.4	C10—C11—H11	120.9
N1—C4—C5	112.61 (13)	C11—C12—C7	120.37 (15)
N1—C4—H4A	109.1	C11—C12—H12	119.8
C5—C4—H4A	109.1	C7—C12—H12	119.8
N1—C4—H4B	109.1		
			
C4—N1—C1—N2	169.47 (13)	O3—C6—C7—C12	−16.0 (2)
C2—N1—C1—N2	−8.8 (2)	N2—C6—C7—C12	161.02 (14)
C4—N1—C1—S1	−8.0 (2)	C12—C7—C8—C9	−1.8 (2)
C2—N1—C1—S1	173.80 (11)	C6—C7—C8—C9	178.89 (14)
C6—N2—C1—N1	134.76 (15)	C7—C8—C9—C10	0.1 (2)
C6—N2—C1—S1	−47.8 (2)	C8—C9—C10—C11	1.4 (3)
C1—N1—C2—C3	89.24 (17)	C8—C9—C10—N3	−178.75 (15)
C4—N1—C2—C3	−89.11 (16)	O5—N3—C10—C11	4.8 (2)
N1—C2—C3—O1	−62.76 (17)	O4—N3—C10—C11	−174.96 (16)
C1—N1—C4—C5	91.30 (17)	O5—N3—C10—C9	−175.05 (17)
C2—N1—C4—C5	−90.33 (16)	O4—N3—C10—C9	5.2 (2)
N1—C4—C5—O2	57.76 (17)	C9—C10—C11—C12	−1.1 (3)
C1—N2—C6—O3	−3.6 (2)	N3—C10—C11—C12	179.10 (15)
C1—N2—C6—C7	179.39 (13)	C10—C11—C12—C7	−0.8 (2)
O3—C6—C7—C8	163.29 (15)	C8—C7—C12—C11	2.2 (2)
N2—C6—C7—C8	−19.7 (2)	C6—C7—C12—C11	−178.47 (14)

### Hydrogen-bond geometry (Å, º)

*Cg*1 is the centroid of the (C7–C12) ring.

**Table:**

*D*—H···*A*	*D*—H	H···*A*	*D*···*A*	*D*—H···*A*
N2—H2*N*···O1	0.87 (1)	1.88 (1)	2.6749 (17)	151 (1)
O1—H1*O*···O2^i^	0.84 (2)	1.87 (2)	2.7075 (17)	176 (2)
O2—H2*O*···S1^ii^	0.84 (1)	2.33 (1)	3.1724 (12)	175 (2)
C2—H2*B*···O3^i^	0.99	2.53	3.2305 (18)	127
C5—H5*A*···S1^iii^	0.99	2.77	3.4915 (17)	130
C8—H8···O3^iv^	0.95	2.36	3.2147 (19)	150
N3—O4···*Cg*1^v^	1.22 (1)	3.63 (1)	3.6927 (16)	83 (1)

Symmetry codes: (i) −*x*+1, *y*+1/2, −*z*+3/2; (ii) *x*−1, *y*, *z*; (iii) −*x*+1, −*y*+1, −*z*+1; (iv) −*x*+2, *y*+1/2, −*z*+3/2; (v) −*x*+2, −*y*+1, −*z*+2.
